# The Effect of Pregabalin on the Minimum Alveolar Concentration of Sevoflurane: A Randomized, Placebo-Controlled, Double-Blind Clinical Trial

**DOI:** 10.3389/fmed.2022.883181

**Published:** 2022-05-03

**Authors:** Johannes Müller, Walter Plöchl, Paul Mühlbacher, Alexandra Graf, Thomas Stimpfl, Thomas Hamp

**Affiliations:** ^1^Division of General Anaesthesia and Intensive Care Medicine, Department of Anaesthesia, Intensive Care and Pain Medicine, Medical University of Vienna, Vienna, Austria; ^2^Center for Medical Statistics, Informatics and Intelligent Systems, Institute for Medical Statistics, Medical University of Vienna, Vienna, Austria; ^3^Department of Laboratory Medicine, Medical University of Vienna, Vienna, Austria

**Keywords:** pregabalin, minimum alveolar concentration (MAC), sevoflurane, anesthesia, depth of anesthesia, premedication before anesthesia

## Abstract

**Background:**

Pregabalin is commonly used perioperatively to reduce post-operative pain and opioid consumption and to prevent the development of chronic pain. It has been shown to reduce anesthetic consumption in balanced anesthesia, but studies investigating its effect on the minimum alveolar concentration (MAC) of volatile anesthetics are lacking. The aim of this study was to investigate the effect of two different doses of pregabalin on the MAC of sevoflurane.

**Methods:**

In a randomized, double-blinded, placebo controlled clinical study, 75 patients were assigned to receive placebo, 300 mg pregabalin, or 150 mg pregabalin, as a capsule 1 h before anesthesia induction with sevoflurane only. After equilibration, the response to skin incision (movement vs. non-movement) was monitored. The MAC was assessed using an up- and down-titration method.

**Results:**

The MAC of sevoflurane was estimated as 2.16% (95% CI, 2.07–2.32%) in the placebo group, 1.44% (95% CI, 1.26–1.70%) in the 300 mg pregabalin group, and 1.81% (95% CI, 1.49–2.13%) in the 150 mg pregabalin group. We therefore report a 33% reduction in the MAC of sevoflurane in the 300 mg pregabalin group as compared to placebo. The MAC of the 150 mg pregabalin group was reduced by 16% as compared to placebo but was not statistically significant.

**Conclusions:**

The administration of 300 mg pregabalin reduced the MAC of sevoflurane by 33%, while the administration of 150 mg pregabalin did not significantly reduce the MAC of sevoflurane. Pregabalin use led to a small reduction in post-operative pain levels but increased side effects in a dose-dependent manner.

## Introduction

Pregabalin is an antiepileptic drug also licensed to treat neuropathic pain and anxiety disorders (1). Pregabalin binds to α2δ subunits of high voltage-activated calcium channels and decreases the release of excitatory neurotransmitters (2). In recent years, it has increasingly been used perioperatively to improve post-operative pain control, reduce post-operative opioid consumption and prevent the development of chronic post-operative pain (3). Although there is ongoing controversy about the clinical benefits vs. risks of its perioperative use, pregabalin is still part of many protocols for multimodal perioperative analgesia (4).

The concept of the minimum alveolar concentration (MAC), which is defined as the volumetric concentration of an inhaled anesthetic that prevents movement in response to a noxious stimulus in 50% of subjects, was introduced more than 50 years ago and remains the most used parameter to guide anesthetic depth during inhalational anesthesia (5). The MAC also enables quantification of the effect of adjunctive drugs on inhalational anesthetics. Despite the widespread use of pregabalin in the perioperative period and sevoflurane being one of the most commonly used inhalational anesthetic agents, studies regarding the effect of pregabalin on the MAC of sevoflurane in humans are lacking. Therefore, the aim of this study was to investigate the effect of pregabalin on the MAC of sevoflurane.

## Methods

This study was performed in accordance with the Declaration of Helsinki, and the CONSORT (Consolidates Standards of Reporting Trials) guidelines were followed during the preparation of this article. We conducted this single-center, prospective, randomized, controlled, double blinded trial between September 2019 and February 2021 at the University Department of Anesthesia, Intensive Care Medicine and Pain Medicine at the Medical University of Vienna, Vienna, Austria. The trial was registered at EudraCT before patient enrolment (EudraCT re. no. 2017-001439-37). Approval by the institutional ethics committee and the regulatory authority was obtained (Ethics Committee of the Medical University of Vienna, Bundesamt für Sicherheit im Gesundheitswesen) before patient enrolment. Patients were included only after written informed consent was obtained.

### Study Population

We recruited adult patients with an American Society of Anesthesiologists physical status of 1–2 scheduled for elective surgery under general anesthesia. To standardize the noxious stimulus, we only included patients undergoing breast surgery, as this usually requires a skin incision of 3–5 cm at the trunk (6). The patients' age was restricted to 30–65 years, as the MAC is relatively uniform in this age group (7). We excluded patients unable to understand the study procedure, patients with a need for sedative or analgesic premedication or a history of chronic pain, patients with a known allergy to one of the medications used in this study, pregnant or breastfeeding patients, and patients in whom inhalational induction of anesthesia was contraindicated.

### Randomization and Blinding

Patients were randomly assigned to receive placebo, 150 or 300 mg pregabalin. Randomization was performed by a study nurse that was not involved in the experimental part of the study at patient enrolment using the online randomization tool provided by our institution (https://www.meduniwien.ac.at/randomizer/).

To ensure blinding of the patients and the investigators, identical capsules containing the study medication or placebo were provided by the pharmacy of the Vienna General Hospital. The study nurse that had performed the randomization administered the capsule according to randomization results 1 h before the surgery. Based on a pseudonymized patient list that was also only accessible to the study nurse they determined the appropriate sevoflurane concentration for the patient and instructed the investigators on which concentration to target. The investigators therefore were not aware of the patients' randomization results or the corresponding study group. After the determination of the skin movements the investigators informed the study nurse, who updated the patient list to include the newest result.

### Anesthesia Induction

Routine anesthetic monitoring, including pulse oximetry, electrocardiography, non-invasive blood pressure, and the bispectral index (BIS monitor A2000 software version 3.3, Aspect Medical Systems, Norwood, MA, USA), was applied in the operating room. Anesthesia was then induced solely by multiple deep inhalation breaths of 8 vol% sevoflurane in pure oxygen (8). A laryngeal mask airway (LMA Supreme Airway, Teleflex Medical Europe Ltd, Dublin, Ireland) was inserted once the BIS value had decreased below 40 and the patients were clinically adequately sedated. We ventilated the patients' lungs with tidal volumes of 6 to 8 ml kg^−1^ at a frequency of 10 to 16 breaths per minute to achieve normocapnia (endtidal CO_2_ between 30 and 40 mmHg). Forced air was applied to the lower limbs to maintain normothermia.

### Determination of MAC

The MAC of an inhalational anesthetic is defined as “the minimum alveolar concentration of an anesthetic that prevents movement in response to a noxious stimulus in 50% of subjects” (5). We used a standardized skin incision for the noxious stimulus and an up- and down-titration method to assess the MAC of sevoflurane in the study groups, as this approach shows the potential to provide reliable data with relatively few patients, as compared to other methods (9). After induction of anesthesia, the sevoflurane concentration was adjusted to reach a predetermined end-tidal sevoflurane concentration, which was held constant for at least 15 min before the skin incision. The sevoflurane concentration was measured using the gas-measuring unit of a Dräger Primus (Dräger Austria GmbH, Vienna, Austria) that was calibrated every 24 hours. In the first patient in each study group, the end-tidal sevoflurane concentration was 1.6 vol%. Before the skin incision was made, one investigator ensured unconsciousness of the patients by calling their name, tapping on their shoulders, and asking them to open their eyes. Next, the surgeon was asked to perform a single incision of 3 to 5 cm and then pause for 1 min before continuation of the operation. The response to the skin incision was counted as positive if the patient exhibited “gross purposeful movement of the head or at least one extremity” within 1 min after the skin incision (6). The response to the skin incision was classified as negative if no such movement occurred within 1 min after the skin incision. Coughing, bucking, and straining were not considered gross purposeful movements. One investigator at the head of the operating table observed the response of the patient's head and upper limbs, and a second investigator observed the response of the lower limbs from the foot of the operating table. The end-tidal sevoflurane concentration for the next patient in each study group was either increased by 0.2 vol% if the previous patient in that group had exhibited a positive response to the skin incision or decreased by 0.2 vol% if the previous patient of that group had exhibited a negative response to the skin incision. 0.2 vol% steps were chosen in order to cover a wider range of sevoflurane doses with a smaller number of patients. Smaller steps might have led to decreased power in case the variability in the observed data was larger than expected in the planning phase.

After the response to the skin incision was determined, patients received further anesthetic management at the discretion of the attending anesthetist based on our departmental standards, which include the administration of opioids (i.e., fentanyl and piritramide) and non-opioid analgesics (i.e., metamizole and paracetamol) as well as medical prophylaxis for post-operative nausea and vomiting (i.e., ondansetron and dexamethasone).

### Secondary Endpoint Parameters

A venous blood sample was taken immediately before the skin incision to determine the serum concentration of pregabalin. Furthermore, we collected BIS values, systolic blood pressure, and heart rate throughout the study period at 2 min intervals until 2 min after the skin incision. The patients were asked about their pain level using a numeric rating scale (min 0, max 10) and the presence of nausea, vomiting, or intraoperative awareness while they were in the recovery area. The site of the incision and the areas of movement were also recorded.

### Measurement of Serum Pregabalin Concentration

Pregabalin concentrations in the serum were assessed with MassTox^®^ TDM Serie A test kits (Chromsystems, Gräfeling, Germany) and liquid chromatography–tandem mass spectrometry (LC–MS/MS) consisting of an LC-20 UFLC (Shimadzu, Kyoto, Japan) and a Triple Quad 4500 (Sciex, Framingham, MA, USA) equipped with a TurboIon Source for electrospray ionization.

### Statistical Analysis

The primary endpoint was the MAC of sevoflurane in the three study groups. The MAC values of the sevoflurane concentration of the three groups were estimated using isotonic regression methods (10–12). To further account for the dependence structure in the data due to the up-and-down design, bootstrap methods were used to construct bias-corrected bootstrap confidence intervals for the MAC as well as the differences in MAC values (13). Within each bootstrap step, resamples where randomly generated (separately for the three groups using sampling with replacement) from the sampling distribution (probability of no-reaction for the observed sevoflurane concentrations) and for each resample, the MAC was calculated using isotonic regression and the difference in MAC between groups was calculated. In total, 5,000 resamples were used to generate the bootstrap-distribution. The bootstrap MAC-differences between groups were estimated as the mean over all bootstrap samples and the confidence interval was estimated using the corresponding percentiles of the bootstrap-distribution. For the two main comparisons of the 150 mg and the 300 mg Pregabalin group to placebo 97.5% confidence intervals (Bonferroni-Correction to apply for multiple testing) for the difference in the MAC values were calculated. The comparison between the 300 mg and 150 mg Pregabalin group was performed as a secondary aim. Furthermore, estimators of the MAC and the corresponding 95% confidence intervals were calculated separately for the three groups.

Differences in the secondary endpoint parameters between the groups, including the baseline characteristics, serum pregabalin concentration, BIS, blood pressure, heart rate, systolic blood pressure, post-operative pain scores, and the perioperative doses of opioids and propofol, were investigated with the Kruskal–Wallis test for independent groups. *P*-values were adjusted for multiplicity using a Bonferroni correction, as there were three groups involved. The *X*^2^-test was used to investigate differences in the ASA physical status score; the use of non-opioid analgesics and antiemetics; and the occurrence of negative side effects, including nausea and vomiting, dizziness, headache, and awareness, between the groups. Adjusted *P* < 0.05 were considered statistically significant. The analyses were performed using R (R: A Language and Environment for Statistical Computing, R Core Team, R Foundation for Statistical Computing, Vienna, Austria 2014, https://www.R-project.org) and SPSS 27 (SPSS Statistics, IBM, Armonk, NY, USA). The data are presented as the mean (standard deviation, SD), minimum-maximum, or number (percentage) unless indicated otherwise.

### Sample Size Determination

For the determination of the sample size, simulation studies were performed. The assumptions of the dose-response curve for the placebo group were based on a previous study (14). Therefore, for the placebo group, the dose-response model formula of Görges et al. with a MAC of 2 with a standard deviation of 0.3 was assumed (10). Since a reduction of the MAC of about 20% was assumed to be clinically relevant, for the treatment groups a MAC of 1.6 with a standard deviation of 0.3 was assumed for the sample size calculations. Due to the two primary comparisons (high and low dose compared to placebo), the 97.5% confidence intervals were calculated (Bonferroni Correction to apply for multiple testing). In each simulation step, the MAC was estimated using isotonic regression and confidence intervals for the difference in MAC between groups were constructed using 1,000 bootstrap samples. 10,000 simulation runs were performed to estimate the power. Under the given assumptions, simulation studies showed a power of 80.8% for a per-group sample size of 22 patients per group. Due to some possible drop-outs, the sample size was fixed with 25 per group.

## Results

**Figure 1:**
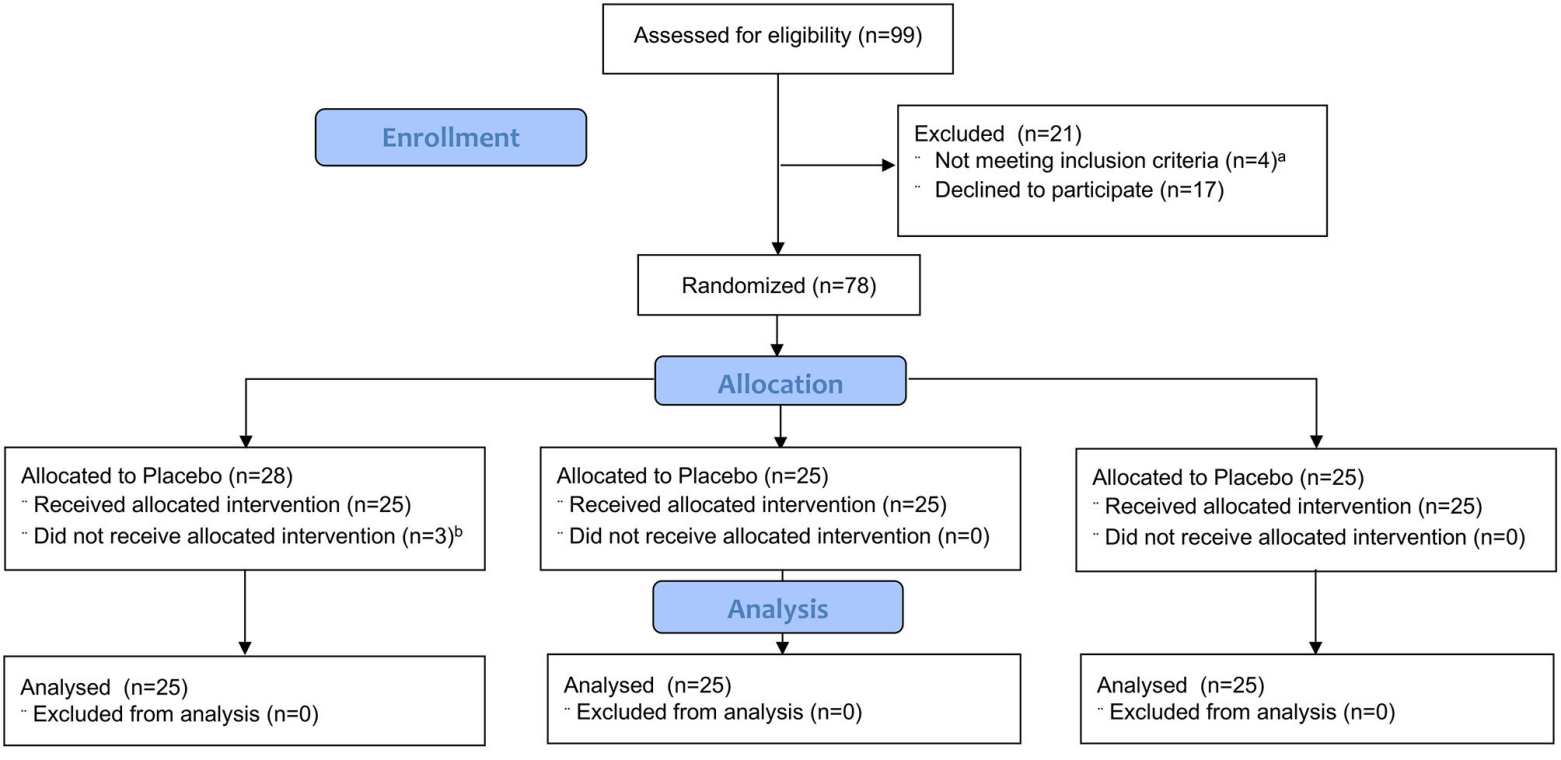
CONSORT flow diagram. CONSORT, Consolidated standards of reporting trials. ^a^Reasons for exclusion: 4 patients received sedative premedication. ^b^Reasons for not receiving allocated intervention: 3 patients developed mild laryngospasm at induction that required intravenous anesthetics.

Seventy-eight female patients were recruited for our study (Figure 1). Three of these patients did not complete the study procedure because they required intravenous anesthetics for the treatment of laryngeal spasms that occurred during induction.

Patient characteristics, morphometric data and the time from the insertion of the laryngeal mask airway to skin incision were similar in all groups (Table 1).

**Table 1:** Subject characteristics and morphometric data.

**Group**	**Placebo (*n* = 25)**	**300 mg pregabalin (*n* = 25)**	**150 mg pregabalin (*n* = 25)**	
Age, years	48 (8), 31–61	47 (8), 31–65	51 (8), 32–65	*P* = 0.193
Height, cm	166 (4), 154–172	167 (7), 155–185	165 (5), 156–176	*P* = 0.612
Weight, kg	70 (10), 52–85	68 (12), 50–97	69 (11), 53–90	*P* = 0.884
BMI, kg m^2^	25 (3), 19–32	25 (4), 19–34	26 (4), 19–34	*P* = 0.562
ASA physical status (*n*)	ASA 1 = 16	ASA 1 = 13	ASA 1 = 14	*P* = 0.683
	ASA 2 = 9	ASA 2 = 12	ASA 2 = 11	
Equilibration time, minutes	21 (7), 15–39	20 (6), 15–41	20 (5), 15–34	*P* = 0.337
Duration of surgery, minutes	74 (44), 19–182	87 (56), 8–225	77 (51), 17–216	*P* = 0.679
Body temperature, °C	36.2 (0.4), 35.4–37.2	36.2 (0.4), 35.6–36.9	36.1 (0.5), 35–36.8	*P* = 0.680

*Data are presented as the mean (standard deviation), minimum-maximum, or absolute number (n)*.

### Primary Outcome

**Figure 2:**
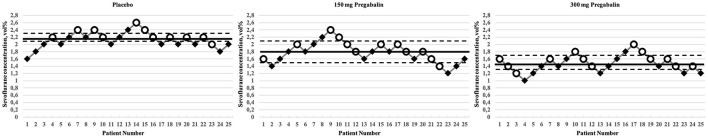
Titration process in the study groups. Circles indicate patients who moved, and rhombi indicate patients who did not move. Solid horizontal lines indicate the bootstrap estimates for the MAC, dashed horizontal lines indicate the 95% confidence intervals. MAC, minimum alveolar concentration.

The bootstrap estimate of the MAC of sevoflurane was 2.16% (95% CI, 2.07–2.32%) in the placebo group, 1.81% (95% CI, 1.49–2.13%) in the 150 mg pregabalin group, and 1.44% (95% CI, 1.26–1.70%) in the 300 mg pregabalin group (Figure 2).

The MAC estimate of sevoflurane in the 300 mg pregabalin group was 33% lower than that in the placebo group. As the confidence intervals of the difference in the MAC values between the placebo group and the 300 mg pregabalin group did not contain 0, this difference in the MAC estimates was statistically significant (97.5% CI for difference: 0.39–1.01%). No significant difference in the MAC estimate was found between the placebo and the 150 mg pregabalin group or the 300 mg pregabalin group and the 150 mg pregabalin group. Table 2 shows the estimates of the MAC of sevoflurane and the corresponding bootstrap confidence intervals to investigate the MAC within the groups as well as the difference between groups.

**Table 2:** MAC estimates, 95% confidence intervals, and differences in the MAC between the study groups.

	**Sample estimates**	**Bootstrap estimates**	**Bootstrap lower CI**	**Bootstrap upper CI**
MAC sevoflurane placebo group, vol%	2.13	2.16	2.07	2.32
MAC sevoflurane 300 mg group, vol%	1.40	1.44	1.26	1.70
MAC sevoflurane 150 mg group, vol%	1.86	1.81	1.49	2.13
Difference from the placebo group −150 mg group, vol%	0.27	0.35	−0.04	0.75
Difference from the placebo group −300 mg group, vol%	0.73	0.72^*^	0.39	1.01
Difference between the 300 and 150 mg groups, vol%	0.46	0.36	−0.09	0.80

* *Indicates a statistically significant difference*.

### Secondary Outcomes

Pregabalin was not detected in the serum of patients in the placebo group. The mean serum pregabalin concentration was 4.2 (SD 1.6) μg ml^−1^ in the 150 mg pregabalin group and 9 (SD 2.8) μg ml^−1^ in the 300 mg pregabalin group (Table 3).

**Table 3:** Serum pregabalin concentration, bispectral index, blood pressure, and heart rate at different time points in the study groups.

**Group**	**Placebo (*n* = 25)**	**150 mg pregabalin (*n* = 25)**	**300 mg pregabalin (*n* = 25)**		**a**	**b**	**c**
Serum pregabalin concentration, μg ml^−1^	0 (0), 0–0	4.2 (1.6), 0–7.0	9 (2.8), 1.2–12.9	*P* < 0.001^*^	<0.001^*^	<0.001^*^	0.001^*^
BIS at incision	38 (9), 24–57	48 (13), 15–70	51 (10), 29–78	*P* < 0.001^*^	*P* = 0.008^*^	*P* < 0.001^*^	*P* = 0.637
Systolic blood pressure before incision, mmHg	101 (11), 84–125	101 (15), 75–141	103 (15), 82–141	*P* = 0.866			
Systolic blood pressure after incision, mmHg	107 (15), 84–150	101 (18), 56–129	104 (20), 75–155	*P* = 0.676			
Heart rate before incision, bpm	65 (9), 49–82	67 (14), 49–100	64 (9), 53–84	*P* = 0.846			
Heart rate after incision, bpm	73 (16), 44–114	69 (17), 44–107	70 (11), 50–88	*P* = 0.897			

*Data are presented as the mean (standard deviation), minimum-maximum; a = placebo vs. 150 mg pregabalin group, b = placebo vs. 300 mg pregabalin-group, c = 150 mg pregabalin vs. 300 mg pregabalin-group*.

* *P < 0.05. BIS, bispectral-index*.

### BIS, Blood Pressure, Heart Rate

The BIS at the time of skin incision was significantly lower in the placebo group than in the 150 mg pregabalin and 300 mg pregabalin groups. No significant differences between the groups were observed regarding systolic blood pressure or heart rate before or after the skin incision (Table 3).

### Post-operative Pain

The mean post-operative pain score reported on the numeric rating scale and the cumulative dose of post-operative piritramide were significantly higher in the placebo group than in both pregabalin groups (Table 4).

**Table 4:** Side effects.

**Group**	**Placebo (*n* = 25)**	**150 mg pregabalin (*n* = 25)**	**300 mg pregabalin (*n* = 25)**		**a**	**b**	**c**
Pain level in the recovery unit, NRS	2.5 (2.2), 0–6	0.7 (1.1), 0–3	1 (1.6), 0–6	*P* = 0.003^*^	*P* = 0.002^*^	*P* = 0.007^*^	*P* = 0.636
Total negative side effects, *n* (percentage)	1 (4)	3 (12)	8 (32)	*P* = 0.001^*^	*P* = 0.001^*^	*P* = 0.006^*^	*P* = 0.009^*^
Nausea and Vomiting, *n* (percentage)	1 (4)	1 (4)	0 (0)	*P* = 0.598			
Dizziness, n (percentage)	0 (0)	0 (0)	8 (32)	*P* < 0.001^*^			
Headache	0 (0)	2 (8)	0 (0)	*P* = 0.128			
Awareness, *n* (percentage)	0 (0)	0 (0)	0 (0)	n.a.			
Cumulative propofol dose intra OP, mg	122 (71), 0–290	122 (54), 50–260	139 (71), 40–290	*P* = 0.694			
Cumulative fentanyl dose intra OP, μg	284 (178), 100–850	245 (116), 100–575	238 (83), 100–350	*P* = 0.832			
Number of patients receiving metamizol intra OP, *n* (percentage)	20 (80)	20 (80)	23 (92)	*P* = 0.409			
Number of patients receiving paracetamol intra OP, *n* (percentage)	1 (4)	0 (0)	1 (4)	*P* = 0.598			
Number of patients receiving diclofenac intra OP, *n* (percentage)	7 (28)	3 (12)	2 (8)	*P* = 0.125			
Number of patients receiving dexamethason intra OP, *n* (percentage)	20 (80)	15 (60)	13 (52)	*P* = 0.105			
Number of patients receiving ondansetron intra OP, *n* (percentage)	13 (52)	12 (48)	12 (48)	*P* = 0.948			
Cumulative piritramid dose in the recovery unit, mg	3.3 (3.1), 0–9	1.4 (2.5), 0–9	1.8 (3), 0–9	*P* = 0.027^*^	0.012^*^	0.037^*^	0.675
Number of patients receiving metamizol in the recovery unit, *n* (percentage)	9 (36)	5 (20)	5 (20)	*P* = 0.324			
Number of patients receiving paracetamol in the recovery unit, *n* (percentage)	5 (20)	1 (4)	2 (8)	*P* = 0.162			
Number of patients receiving diclofenac in the recovery unit, *n* (percentage)	5 (20)	1 (4)	1 (4)	*P* = 0.080			

*Data are presented as the mean (standard deviation), minimum-maximum; or absolute number (percentage); a = placebo vs. 150 mg pregabalin-group, b = placebo vs. 300 mg pregabalin group, c = 150 mg pregabalin vs. 300 mg pregabalin-group*.

* *P < 0.05*.

### Side Effects

Patients in the 150 mg pregabalin group (12%) and the 300 mg pregabalin group (32%) reported negative side effects, such as nausea and vomiting, dizziness and headache, more frequently than patients in the placebo group (4%).

None of the patients in any group reported an event of intraoperative awareness.

A summary of the secondary outcome parameters is provided in Table 4.

## Discussion

In this prospective, randomized, controlled, double-blinded study, we assessed the effect of two different doses of pregabalin on the MAC of sevoflurane in female ASA 1 and 2 patients undergoing elective surgery. We found a 33% reduction in the MAC at a dose of 300 mg pregabalin compared to placebo but there was no statistically significant reduction at a dose of 150 mg.

In neuropathic pain, pregabalin seems to “impair the trafficking of α2δ-1 to presynaptic terminals of dorsal root ganglion neurons, which would reduce Ca^2+^ influx and transmitter release in the spinal cord and subsequently reduce spinal sensitization” (15). Although acute pain is caused by different mechanisms than neuropathic pain, pregabalin has been shown to reduce acute pain in various animal models (16, 17). Furthermore, pregabalin has been reported to decrease the isoflurane and sevoflurane requirements during balanced anesthesia (18, 19). There is evidence that sevoflurane acts on gamma-aminobutyric acid-receptors, which increase the release of inhibitory neurotransmitters (20, 21). Similar pharmacological effects of pregabalin and sevoflurane in the context of general anesthesia might therefore be explained by the overlap of pharmacodynamic principles.

Our results are in line with these previous reports indicating an anesthesia-enhancing effect of pregabalin. However, the endpoint of MAC testing (gross purposeful movements in response to a painful stimulus) might be affected not only by pain perception but also by the motor response to painful stimulation. Our study design did not allow us to discriminate between these possible mechanisms, and additional studies are necessary to elucidate the underlying mechanism of the observed MAC reduction.

Various guidelines recommend the use of depth of anesthesia monitors such as the BIS to guide the depth of anesthesia in certain patient groups (22). We found that for the same endpoint of 50% of the patients moving and 50% not moving in response to skin incision, the BIS values were significantly lower in the placebo group than in the pregabalin group. This is not surprising, as the average sevoflurane level in the placebo groups was higher than that in the pregabalin groups. It seems that pregabalin does not enhance immobility *via* cerebral depressing effects, which should also have decreased the BIS. As inhalational anesthetics produce immobility mainly by acting on the spinal cord, we speculate that pregabalin enhances immobility at the same level, especially since we observed the same degree of immobility at higher BIS levels with pregabalin (23).

We chose to use doses of 300 mg and 150 mg pregabalin, as these doses are most commonly used in the perioperative setting, and found a statistically significant reduction in the MAC in the 300 mg pregabalin group but not in the 150 mg pregabalin group (3). Several explanations for this finding, such as an increased interindividual variation in the MAC caused by variations in the serum pregabalin concentrations or the effect of the initial sevoflurane concentration in this up-and-down design, are possible (24). Most likely, our study was just underpowered to detect minor differences in the MAC, but the clinical relevance of such minor differences in the MAC remains questionable. Nevertheless, given that 150 mg pregabalin is associated with fewer unwanted side effects and shows the potential to reduce the MAC of sevoflurane as well as post-operative pain and opioid consumption, future studies should focus on dosages lower than 300 mg.

Recently, the perioperative usefulness of pregabalin has been questioned, as its effect on post-operative and chronic pain appears to be minimal, and side effects seem to be common (4). Our results suggest that pregabalin reduces post-operative pain and opioid consumption in general but not in a dose-dependent manner. At the same time, side effects were significantly increased with higher doses of pregabalin. In our study, anesthetic procedures that followed the initial skin incision were not standardized. We chose this approach to be able to provide individualized patient care to guarantee the best medical outcome. However, this means that the secondary outcome parameters that refer to post-operative pain or opioid consumption should only be considered in terms of hypothesis generating.

In addition to the limitations mentioned above, we need to mention that only female patients were included in this study. While there is no evidence that the MAC of conventional inhalational anesthetics is affected by sex, we cannot rule out that the effects of pregabalin differ between men and women (25). However, as only female patients undergoing breast surgery were investigated, our study group contained a very uniform patient population, limiting interindividual variation due to surgery or sex.

In conclusion, the preoperative administration of 300 mg pregabalin reduced the MAC of sevoflurane by 33%, while the administration of 150 mg pregabalin did not significantly reduce the MAC. Pregabalin use led to a small reduction in post-operative pain levels but increased side effects in a dose-dependent manner.

## Data Availability Statement

The raw data supporting the conclusions of this article will be made available by the authors, without undue reservation.

## Ethics Statement

The studies involving human participants were reviewed and approved by Ethikkommission der Medizinischen Universität Wien, Borschkegasse 8b/E06, 1090 Wien. The patients/participants provided their written informed consent to participate in this study.

## Author Contributions

JM generated the concept, administered the project, conducted the investigation, provided resources, and wrote the original draft of the manuscript. WP generated the concept, provided resources and guidance in conducting the investigation, and edited the manuscript. PM conducted the investigation and edited the manuscript. AG generated the concept, provided statistical planing, conducted the formal analysis, and edited the manuscript. TS conducted the formal and analytical analysis and edited the manuscript. TH generated the concept, provided resources, wrote the original draft of the manuscript, edited the manuscript, and supervized the project as a whole. All authors contributed to the article and approved the submitted version.

## Funding

This study received funding from Chiesi Pharmaceuticals GmbH, Austria. The funder was not involved in the study design, collection, analysis, interpretation of data, the writing of this article, or the decision to submit it for publication.

## Conflict of Interest

The authors declare that the research was conducted in the absence of any commercial or financial relationships that could be construed as a potential conflict of interest.

## Publisher's Note

All claims expressed in this article are solely those of the authors and do not necessarily represent those of their affiliated organizations, or those of the publisher, the editors and the reviewers. Any product that may be evaluated in this article, or claim that may be made by its manufacturer, is not guaranteed or endorsed by the publisher.
